# A retrospective claims analysis of combination therapy in the treatment of adult attention-deficit/hyperactivity disorder (ADHD)

**DOI:** 10.1186/1472-6963-9-95

**Published:** 2009-06-08

**Authors:** Gerhardt M Pohl, David L Van Brunt, Wenyu Ye, William W Stoops, Joseph A Johnston

**Affiliations:** 1 Lilly USA, LLC, Indianapolis, IN, USA; 2 Eli Lilly and Company, Indianapolis, IN, USA; 3 Lilly Inter-Continental Information Sciences (ICIS), Sydney, Australia; 4 University of Kentucky College of Medicine, Department of Behavioral Science, Lexington, KY, USA

## Abstract

**Background:**

Combination therapy in managing psychiatric disorders is not uncommon. While combination therapy has been documented for depression and schizophrenia, little is known about combination therapy practices in managing attention-deficit/hyperactivity disorder (ADHD). This study seeks to quantify the combination use of ADHD medications and to understand predictors of combination therapy.

**Methods:**

Prescription dispensing events were drawn from a U.S. national claims database including over 80 managed-care plans. Patients studied were age 18 or over with at least 1 medical claim with a diagnosis of ADHD (International Classification of Diseases, Ninth Revision, Clinical Modification [ICD-9-CM] code 314.0), a pharmacy claim for ADHD medication during the study period July2003 to June2004, and continuous enrollment 6 months prior to and throughout the study period. Dispensing events were grouped into 6 categories: atomoxetine (ATX), long-acting stimulants (LAS), intermediate-acting stimulants (IAS), short-acting stimulants (SAS), bupropion (BUP), and Alpha-2 Adrenergic Agonists (A2A). Events were assigned to calendar months, and months with combined use from multiple categories within patient were identified. Predictors of combination therapy for LAS and for ATX were modeled for patients covered by commercial plans using logistic regression in a generalized estimating equations framework to adjust for within-patient correlation between months of observation. Factors included age, gender, presence of the hyperactive component of ADHD, prior diagnoses for psychiatric disorders, claims history of recent psychiatric visit, insurance plan type, and geographic region.

**Results:**

There were 18,609 patients identified representing a total of 11,886 months of therapy with ATX; 40,949 months with LAS; 13,622 months with IAS; 38,141 months with SAS; 22,087 months with BUP; and 1,916 months with A2A. Combination therapy was present in 19.7% of continuing months (months after the first month of therapy) for ATX, 21.0% for LAS, 27.4% for IAS, 23.1% for SAS, 36.9% for BUP, and 53.0% for A2A.

For patients receiving LAS, being age 25–44 or age 45 and older versus being 18–24 years old, seeing a psychiatrist, having comorbid depression, or having point-of-service coverage versus a Health Maintenance Organization (HMO) resulted in odds ratios significantly greater than 1, representing increased likelihood for combination therapy in managing adult ADHD.

For patients receiving ATX, being age 25–44 or age 45 and older versus being 18–24 years old, seeing a psychiatrist, having a hyperactive component to ADHD, or having comorbid depression resulted in odds ratios significantly greater than 1, representing increased likelihood for combination therapy in managing adult ADHD.

**Conclusion:**

ATX and LAS are the most likely drugs to be used as monotherapy. Factors predicting combination use were similar for months in which ATX was used and for months in which LAS was used except that a hyperactive component to ADHD predicted increased combination use for ATX but not for LAS.

## Background

Attention-deficit/hyperactivity disorder (ADHD) is a psychiatric disorder that originates in childhood and often continues into adulthood. It has been estimated that between 3% and 10% of all children are affected by ADHD and that 33% to 66% of these children have ADHD symptoms into adulthood [1]. The disorder is characterized by persistent inattention, impulsive hyperactivity, or both [2] and is often comorbid with a number of other psychiatric disorders [3-5]. These symptoms lead to a number of impairments, with adults diagnosed with ADHD being less likely to graduate high school or college, hold employment, or report having life satisfaction [6].

Medications are commonly used to manage ADHD. Most often used are stimulants that interact with brain monoamine systems. These include amphetamines (Dexedrine or Adderall) and methylphenidate (Concerta, Ritalin, or Focalin). Nonstimulant medications include atomoxetine (Strattera) and bupropion (Wellbutrin) [7]. Stimulants like amphetamines and methylphenidate are highly effective pharmacological treatments for ADHD and are used as "first-line" agents for managing this disorder [7,8]. Atomoxetine also improves ADHD symptoms, but may not be as fast acting or effective as amphetamines or methylphenidate for addressing acute behavioral concerns [7,9-11]. Although these medications have proven effective in managing ADHD to varying degrees, there are some patients who do not respond favorably to any single medication and may require a combination of treatments to manage ADHD [12,13].

Polypharmacy (combination therapy) in managing psychiatric disorders is not uncommon [14-17]. Indeed, patients with treatment-resistant depression or schizophrenia often receive combinations of medications to alleviate their symptoms. In 1 study, for example, patients that had greater severity of symptoms were more likely to be treated with multiple medications upon discharge from an inpatient psychiatric unit [14]. While combination therapy has been documented for depression and schizophrenia, little is known about combination therapy practices in managing ADHD. Results of a recent chart review do indicate that combination therapy for adult ADHD is used to prolong therapeutic effects or manage primary medication side effects [18].

The purpose of the present study is to examine the prevalence of combination therapy in adults with ADHD using a retrospective claims analysis of data from a large data warehouse. These data are also used to determine which demographic or utilization patterns, if any, predict combination therapy in adult ADHD patients receiving either long-acting stimulants or atomoxetine. We focus on these medications as they represent the most comparable treatments in terms of duration of action and represent the newest additions and possibly most costly therapies. Note that our perspective is not clinical. We make no claims or recommendations regarding the effectiveness of combination therapy or the tradeoffs in terms of cost or increased exposure to potential side effects of medication. Instead, our research is an examination from the health systems perspective, where combination use may be seen as duplicative or an indication that existing resources are not being used to maximal effect.

## Methods

This study was a retrospective claims analysis of data obtained from the commercially available PharMetrics, Inc. data warehouse on October 6, 2005. Medication usage was analyzed for the period from July 2003 to June 2004. These data reflect U.S. national medical and pharmacy claims covering more than 80 managed-care organizations and 60 million beneficiaries. All data received had been previously de-identified so as to maintain the confidentiality of patients. This study was conducted in compliance to all requirements of the Health Insurance Portability and Accountability Act (HIPAA).

The initial subset drawn included all patients (2,041,434) with at least 1 medical claim with a diagnosis of ADHD (ICD9-CM code 314.0). We narrowed the focus to patients who were enrolled 6 months prior to and throughout the study period (435,536: 21.3%). From these, we identified patients 18 years or older in 2003 (305,469: 70.1%). Finally, we determined which of those patients had a prescription for an ADHD medication during the study period (18,609: 6.1%). This was the basis for our final analysis group. It is important to note that while demographics are given for the individuals included in the study, the unit of analysis is patient-months during which individuals received a prescription for an ADHD medication.

Data were analyzed as patient-months of treatment on any given class of medication, and each calendar month of treatment was characterized as being associated with either monotherapy or combination use of medications. Patient-level claims were assigned based on the date of service and days' supply. Supplies of less than or equal to 31 days were assigned to a single month; supplies of 32 to 60 days, to 2 months; and 61 to 90, to 3 months and so on. The starting month was taken to be the month of the claim if a greater portion of the supply was available in that month than in the last succeeding month covered by the claim. Otherwise, the starting month was shifted to the month immediately following the claim date. In addition, to focus on long-term combination therapy rather than details of transitional management (tapering, unused leftover supply, etc.), the first month of each continuous treatment episode was excluded from analysis.

As shown in Table 1, the analysis divided medications used to treat ADHD into 6 classes: long-acting stimulants (LAS), intermediate-acting stimulants (IAS), short-acting stimulants (SAS), atomoxetine (ATX), buproprion (BUP), and alpha-2 adrenergic agonists (A2A). Although BUP and A2A are not indicated for ADHD, they have been used "off-label" to treat ADHD [7,19] and were included in this analysis.

**Table 1:** ADHD Drug Classes as Defined in This Study.

Drug Class	Medications Included
Atomoxetine	Strattera
	
Long-Acting Stimulants (LAS)	Ritalin LA
	Metadate CD
	Concerta
	Focalin XR
	Adderall XR
	*Generic pemoline*
	Cylert
	
Intermediate-acting Stimulants (IAS)	*Generic methylphenidate (ER/SR/CR/SA)*
	Ritalin SR
	Metadate ER
	Methylin ER
	*Generic amphetamines (CR)*
	Adderall
	Dexedrine spansules
	Desoxyn
	
Short-Acting Stimulants (SAS)	*Generic methylphenidate (short-acting)*
	Ritalin
	Methylin
	Focalin
	*Generic amphetamines*
	Dexedrine
	Dextrostat
	Dexampex
	Biphetamine
	Provigil
	
Buproprion (BUP)	*Generic bupropion (SR/ER)*
	*Generic bupropion*
	Wellbutrin
	Wellbutrin SR
	Wellbutrin XL
	Zyban
	
Alpha-2 Adrenergic Agonists (A2A)	Clonidine
	Guanfacine

We determined the predictors of combination therapy separately for LAS and ATX in those patients covered by commercial plans. To adjust for correlation within the months of treatment in the same patient, logistic regression was performed in a general estimating equations framework with exchangeable covariance structure between months within patient. Factors included in the models were age, gender, presence of hyperactive component to the ADHD, prior claim diagnoses for various psychiatric disorders, claims history of prior psychiatric visit, insurance plan type, and geographic region. Diagnoses were considered positive if seen in 2 claims on separate days in the 6 months prior to study period, and psychiatric service use was considered positive if seen in 1 claim prior to study period.

Analyses were performed using the SAS^® ^system version 8.02 for Unix (Cary, North Carolina) with predictive models fit using PROC GENMOD. Confidence intervals are reported at the 95% level.

## Results

### Demographics

Demographic characteristics of patients contributing prescription events are presented in Table 2.

**Table 2:** Demographic Characteristics of Patients (N = 18,609) Included in This Analysis.

**Variable**	**Percentage**
**Age in Years**	
18–24	38.33%
25–44	34.99%
45 and older	26.68%
**Gender**	
Male	54.44%
Female	45.56%
**Prior Claim Diagnoses (ICD9-CM codes)**	
ADHD Hyperactive Type (314.01)	30.91%
Psychotic Disorders (295, 297, 298)	1.30%
**Bipolar/Mania**	
(296.0, 296.1, 296.4, 296.5 – 8,301.13)	5.63%
Depression (296.2, 296.3, 300.4, 311)	38.48%
Anxiety States (300)	19.85%
Personality Disorders (301)	1.98%
Tics/Tourette's (307.2)	0.40%
Substance Abuse/Dependence (303 – 305)	10.80%
Eating Disorders (307.1, 307.50 – 307.51)	0.87%
Psychiatric Visit in Prior 6 Months	24.88%
**Payer Type**	
Commercial Plan	92.04%
Medicaid	2.72%
Self-Insured	2.91%
Other/Unknown	2.33%
**Plan Type**	
HMO	49.13%
Preferred Provider	31.65%
Point of Service	12.66%
Indemnity	4.33%
Other/Unknown	2.24%
**Region**	
Midwest	56.08%
South	25.43%
East	12.14%
West	6.35%

### Medications and Medication Combinations for ADHD

Total patient months of LAS, IAS, or SAS treatment were 40,949; 13,622; and 38,141, respectively. Total patient months of the nonstimulants – ATX, BUP, or A2A – were 11,886; 22,087; and 1,916, respectively. Of these, 34,239; 11,200; 31,239; 8,141; 18,318; and 1,639 were months following the first months of usage for each medication class (Table 3).

**Table 3:** Percent of Months of Treatment in which Multiple ADHD Medications were Prescribed

Drug Class (number of months of therapy excluding first months)	LAS	IAS	SAS	ATX	BUP	A2A	Any other ADHD medication
LAS (34,239)	79.0	1.0	9.5	1.9	9.3	1.1	21.0
IAS (11,200)	2.5	72.6	17.1	1.1	8.4	0.8	27.4
SAS (31,239)	9.1	5.9	76.9	1.5	7.8	1.0	23.1
ATX (8,141)	6.7	1.0	4.7	80.3	8.6	1.0	19.7
BUP (18,318)	17.0	5.0	13.5	4.4	63.1	1.2	36.9
A2A (1,639)	22.3	5.5	18.3	5.7	12.2	47.0	53.0

Rows represent therapy of focus. Percents represent breakout of proportion of months in combination with other therapies. Numbers on the diagonal from top left to lower right represent number of months on monotherapy on Long-Acting Stimulants (LAS), Intermediate-Acting Stimulants (IAS), Short-Acting Stimulants (SAS), Atomoxetine (ATX), Bupropion (BUP), and Alpha-2 Adrenergic Agonists (A2A). Row sums across individual drug classes may be larger than the value shown for combination with any class due to more than 2 classes being prescribed in combination.

When analyzed in terms of combination months (i.e., non-first months in which patients received more than 1 medication for ADHD), patients received medication combinations 21.0% of months for LAS, 27.4% of months for IAS, and 23.1% of months for SAS (Table 3). Patients received medication combinations 19.7% of months for ATX, 36.9% of months for BUP, and 53.0% of months for A2A.

Stimulants and ATX appeared in combination with BUP approximately 8–9% of months. Excluding BUP, ATX was used most frequently in combination with LAS (6.7% of months); LAS, most frequently with SAS (9.5% of months); IAS, with SAS (17.1% of months); and SAS with LAS (9.1% of months).

### Predictors of Combination Therapy in Adult ADHD

**Figure 1:**
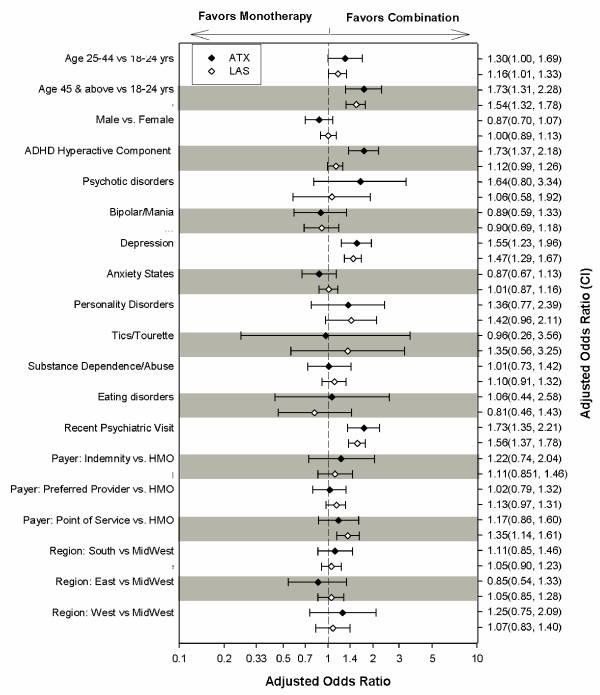
**Adjusted Odds Ratio for 2 Models, Long-Acting Stimulants and Atomoxetine**. Adjusted odds ratios (confidence intervals) for combination therapy use in 2 models, long-acting stimulants (LAS) and atomoxetine (ATX) in adults with ADHD.

Adjusted odds ratios for various factors predicting whether a given month of therapy would be a month in which multiple medications were received are presented in Figure 1. Note while interpreting the 2 models, that the ATX model is based on a much smaller sample size and, therefore, has much broader confidence intervals than the LAS model. Caution should, therefore, be exercised in interpreting factors that are significant only in the LAS model but not the ATX model.

For patients receiving LAS, being age 25–44 or age 45 and older versus being 18–24 years old, seeing a psychiatrist, having comorbid depression, or having point of service coverage versus a Health Maintenance Organization (HMO) resulted in odds ratios significantly greater than 1, representing increased likelihood for combination therapy in managing adult ADHD.

For patients receiving ATX, being age 25–44 or age 45 and older versus being 18–24 years old, seeing a psychiatrist, having a hyperactive component to ADHD, or having comorbid depression resulted in odds ratios significantly greater than 1, representing increased likelihood for combination therapy in managing adult ADHD.

## Discussion

The results of the present study indicate that combination therapy in adult ADHD is not uncommon. Combination therapy was present in more than 20% of the months of therapy represented in this sample. Approximately 8–9% of the combination therapy of stimulants and of ATX was with BUP. This may not represent true combination therapy for treatment of ADHD but may rather reflect treatment of comorbid conditions, particularly depression.

Combination therapy was least common in patients prescribed LAS and in patients prescribed ATX. For patients prescribed BUP or A2A, combination therapy was much more common. Interestingly, for patients prescribed BUP and A2A, the supplemental medications were most often stimulants.

The finding that combination therapy is not as common for patients prescribed stimulants relative to BUP and A2A, despite that these medications were the class most often prescribed is consistent with the use of these indicated drugs as "first line" agents for adult ADHD [7]. BUP and A2A, on the other hand, may well be used in this adult sample to treat conditions other than ADHD, and their presence here may in part reflect psychiatric comorbidities in addition to ADHD treatment. Interestingly, despite debates [7-10] over its efficacy, ATX was not used more often in combination therapy than the stimulants.

IAS showed markedly more combination use with SAS than either LAS or ATX. This may reflect the more limited duration of action of IAS being insufficient to maintain adequate ADHD symptom control throughout the entire day.

To better understand the factors that contributed to combination therapy in patients prescribed long-acting ADHD medications, specifically LAS and ATX, we calculated odds ratios for combination therapy from patient characteristics. A number of these factors were associated with combination therapy. These included being older, having psychiatric care, and having comorbid depression. The association between psychiatric care and combination therapy is not surprising given that patients with more severe or treatment refractory disease are more likely to be referred for psychiatric care after having failed 1 or more simpler treatment regimens.

In contrast to previous models where the decision to initiate ATX rather than LAS was associated with claims histories suggesting high comorbidities and clinical complexity [20], the pattern of factors predicting rates of combination therapy were mostly similar for both ATX and LAS. The most salient difference between the current models was the hyperactive component of ADHD. Hyperactive component was not a significant predictor of combination therapy for LAS, despite the very large sample size and tighter confidence interval. The hyperactive component was significantly associated with increased combination therapy for ATX. While the prevalence and significance of hyperactive symptoms in adults is unclear, there is some evidence [21] that hyperactive symptoms in children with ADHD may be less likely to persist into adulthood than inattentive symptoms. Another important finding is that patients with HMO insurance plans rather than point-of-service coverage were less likely to receive additional medications for ADHD with LAS. The reason for this difference is unknown, but could indicate that HMOs are more likely to discourage the use of combination therapy in an effort to reduce costs.

As described above, combination therapy in adult psychiatry is not uncommon and is fairly well documented in depression and schizophrenia [14-17]. Combination therapy is not as well understood in patients diagnosed with adult ADHD, although the results of a recent chart review [18] and present findings indicate that it is fairly prevalent and varies depending upon the medications prescribed. Regardless of the diagnosis, combination therapy should be reserved for severe cases or treatment-resistant patients. The results of this study suggest that the medications used most often in monotherapy are LAS and ATX. Using a single medication is beneficial to patients for both physical (e.g., having fewer side effects from 1 medication versus multiple medications) and financial (e.g., cost of 1 versus multiple medications) reasons.

The management of patients with adult ADHD can be complex. In making treatment decisions, clinicians must evaluate the unique characteristics of patients as well as the distinct benefits and risks of different treatment options. For example, although stimulants are very effective in treating ADHD, they have significant abuse potential [7,19]. In patients with histories of substance abuse or dependence (or who may live with those who do), diversion potential becomes a concern and may influence treatment selection. On the other hand, in a randomized, placebo-controlled, head-to-head trial of atomoxetine versus osmotically-released methylphenidate, Newcorn and colleagues (2008) concluded that while patients in the atomoxetine group (n = 222) and the osmotically-released methylphenidate group (n = 220) both showed significant superiority to placebo on the ADHD-RS primary endpoint measure after 6 weeks, clinical response to osmotically-released methylphenidate was superior to the response to atomoxetine (56% vs. 45%, respectively) [22]. Further, although medications can control the core symptoms of ADHD, the use of cognitive or behavioral therapies is also beneficial in managing this disorder [23]. Future research should examine the influence of these therapies on the need for combination therapy, or lack thereof, in adult ADHD patients.

## Limitations

Although these findings provide initial data on combination therapy in adult ADHD, several limitations need to be acknowledged. Claims data are collected for administrative purposes and cannot be considered as accurate or reliable as data collected for a specific scientific purpose. Second, the provider type "psychiatrist" is imputed within the PharMetrics database and may include some psychologists and social workers billing for services typical of psychiatrists. The attribution of a claims history of prior psychiatric visit is, therefore, somewhat imprecise. Third, formulary status, plan restrictions, and tier status were not known and may have influenced the associations described here. Fourth, although these data indicate that LAS and ATX are least likely to be associated with combination therapy in ADHD, direct evidence of effectiveness (i.e., reduction in ADHD symptomatology) is not available in this dataset. Fifth, from the perspective of generalizability, patients from the Midwest and South are overrepresented in this sample, so care should be taken in generalizing too broadly. Finally, although the sample was restricted to patients with a previous claim diagnosis of ADHD, one is not able to conclusively determine the reason for each individual pharmaceutical claim.

## Conclusion

This study suggests that only a small percentage, 6.1% of the adult patients with a history of ADHD, receive any pharmacological treatment during the course of a year. LAS and ATX are the most common pharmacological monotherapies in the treatment of adult ADHD. IAS and SAS were used more frequently in combination with other medications while BUP (an atypical antidepressant) and A2A (antihypertensives that are also used on occasion to promote sleep) were most often to be used in combination with other medications. Factors predicting combination use (personal, clinical, and health care delivery factors) were similar for months in which ATX was used and for months in which LAS were used, except that a hyperactive component to ADHD predicted increased combination use for ATX but not for LAS. Further research is needed to better understand the motivations for combination therapy in managing adult ADHD, particularly in terms of symptom outcomes and nonpharmacological treatments used for ADHD.

## Competing interests

Of the 5 authors, 4 are employees of Eli Lilly and Company, and the fifth author is a consultant to Eli Lilly and Company.

## Authors' contributions

GMP contributed to the design, analysis, interpretation, writing, and review of the manuscript. DVB contributed to the design, interpretation, writing, and review of the manuscript. WY contributed to the analysis, interpretation, and review of the manuscript. WWS contributed to the interpretation, writing, and review of the manuscript. JAJ contributed to the design, interpretation, and review of the manuscript. All authors read and approved the final manuscript.

## Pre-publication history

The pre-publication history for this paper can be accessed here:


